# Suppressed plasmablast responses in febrile infants, including children with Kawasaki disease

**DOI:** 10.1371/journal.pone.0193539

**Published:** 2018-03-26

**Authors:** Meghan Martin, Brian H. Wrotniak, Mark Hicar

**Affiliations:** Department of Pediatrics, University at Buffalo School of Medicine and Biomedical Sciences, Buffalo, NY, United States of America; University of North Carolina at Chapel Hill School of Medicine, UNITED STATES

## Abstract

**Background:**

Kawasaki disease (KD), the leading cause of acquired heart disease in children, primarily affects infants and toddlers. Investigations on immune responses during KD are hampered by a limited understanding of normal immune responses in these ages. It’s well known that Infants have poorer vaccine responses and difficulty with maintaining prolonged serum immunity, but there are few studies on human infants detailing immune deficiencies. Limited studies propose an inability to maintain life-long bone marrow plasma cells. Plasmablasts are a transitional cell form of B cells that lead to long-term Plasma cells. Plasmablasts levels rise in the peripheral blood after exposure to a foreign antigen. In adult studies, these responses are both temporally and functionally well characterized. To date, there have been few studies on plasmablasts in the predominant age range of KD.

**Methods:**

Children presenting to an urban pediatric emergency room undergoing laboratory evaluation, who had concern of KD or had fever and symptoms overlapping those of KD, were recruited. Peripheral blood mononuclear cells were isolated and evaluated utilizing flow cytometry with specific B cell markers from 18 KD subjects and 69 febrile controls.

**Results:**

Plasmablast numbers and temporal formation are similar between infectious disease controls and KD subjects. In both groups, infants have diminished plasmablast responses compared to older children.

**Conclusion:**

In this single-time point survey, infants have a blunted peripheral plasmablast response. Overall, similar plasmablast responses in KD and controls support an infectious disease relationship to KD. Future time-course studies of plasmablasts in infants are warranted as this phenomenon may contribute to observed immune responses in this age group.

## Introduction

Kawasaki Disease (KD) is the leading cause of acquired heart disease in children [1]. Treatment with intravenous immunoglobulin (IVIG) reduces the incidence of coronary artery aneurysms from near a quarter of untreated cases to roughly 10-fold less [1–6]. The long-term effects of childhood KD on adult cardiovascular health remains unclear [7], however, numerous studies point to higher risk of cardiovascular issues and ischemic heart disease in adults [8–11]. Although, the yearly incidence in the United States is 9–19 cases per 100,000 children [12], it is nearly 10-fold higher in Japan, and evidence is that this is increasing over time [13].

KD classically presents as a minimum of five days of fever with conjunctivitis, singular lymph node swelling, oral mucous membrane inflammation, peripheral extremity swelling, and rash [1, 4, 6, 14]. Four of five criteria define classic KD, however, incomplete presentations with fewer symptoms can also result in coronary artery abnormalities [3]. Laboratory values such as leukocytosis, anemia, alanine aminotransferase (ALT) elevation, late thrombocytosis, urethritis, or hypoalbuminemia [2] can be useful to diagnose KD, particularly for incomplete forms [3]. Unfortunately, there is no specific and sensitive test to confirm either the KD diagnosis or to distinguish those that will go on to have aneurysms.

While the cause of KD is unclear [6, 15, 16], seasonality, periodic ‘outbreaks’, the peak age range in early childhood, and clinical symptoms similar to other infectious agents all support an infectious disease as a cause [14]. Diagnosis of KD prior to six months of age is rare and the normal peak in toddlers generally suggests a protective role of maternal antibodies during early development [17]. Recent studies show a lower incidence in breastfed infants further supporting protection by antibodies [18]. Other data support a role of B cell responses in the pathogenesis of KD. B cells are “activated” (CD69+) and downregulation of BCR receptor signaling occurs in Kawasaki disease [19]. Additionally, genome wide association studies implicate CD40 signaling and the involvement of the B Lymphoid Tyrosine Kinase [20, 21] in KD. If KD is caused by an acute infection, studying the acute changes to the B cell populations can be very informative.

During an acute infection, both naïve B cells and memory B cells are stimulated to form plasmablasts: B cells transitioning to plasma cells that circulate in the peripheral blood cell compartment [22, 23] recognized by surface markers of CD19 (B cell marker), downregulation of CD20, and high levels of CD27 and CD38 [1, 24]. In comparison to the general circulating B cell population, plasmablasts are enriched for B cells that contain infection-specific antibodies [25, 26]. This is variable as some studies show massive and high enrichment of plasmablasts targeting the antigen of interest [27, 28], while other studies show polyspecificity of the plasmablast population and limited enrichment [29–32]. Immunization studies in adults (tetanus [33], influenza [24], and rabies [34]) show plasmablast have more more consistent enrichment for specific antibodies, temporally peak 5–10 days after immunization, and are predictive of later sero-immunity [35]. Elevated circulating peripheral plasmablasts are not specific to infections, as they are elevated in a number of autoimmune disease and their levels correlate to disease flares [36]. Although certain infections, such as dengue virus, may set off exceedingly high plasmablast levels [37], plasmablast levels tend to be significantly higher in autoimmune conditions than levels achieved during vaccination or post-infection. This excessive circulating plasmablast response seen corresponds to flaring of the underlying inflammatory disease, and specifically correlates with CRP level in studies on ulcerative colitis [37, 38] and IGG4 related disease [39, 40].

Kawasaki disease is most prevelent, from six months to six years of age, at a time when the immune system is still developing. It is known that infants have poorer responses to infections and vaccinations [41], however, thorough study of plasmablast responses in the predominant age of KD presentation has not been performed. Post- meningococcal vaccine plasmablast responses have been evaluated, and show that infant responses [42] are diminished compared to adult levels [43]. Other infections or vaccinations have not included timecourse evaluation of Plasmablast cells. In this study, we sought to characterize the plasmablast responses of febrile children, including a subset with KD, in the age range of peak KD incidence to provide the basis for future studies on specific plasmablast responses in KD children.

## Methods

### Study design

This is a single site cross-sectional study of children with fever. Children nine months (chosen for safety considerations) to six years of age presenting to the Emergency Department of Women and Children’s Hospital of Buffalo from March 2014 to May 2016 were screened to determine if they met enrollment criteria. Eligible patients met the following criteria: fever of 38.3°C or above prior to presentation, had a planned blood draw as part of the evaluation of their illness, and one of the following symptoms: rash, mucous membrane changes, extremity changes, conjunctivitis or a single isolated enlarged lymph node. Children admitted or transferred from outside facilities with the specific concern for KD were also enrolled regardless of other criteria. These inclusion criteria captured KD children and a selection of febrile controls. Children with KD in this study are defined as those enrolled who were diagnosed by both primary team and Infectious disease (ID) consultant as having clinical KD and who underwent IVIG treatment after their initial blood draw. Other specific diagnoses were defined on clinical criteria alone, or considered confirmed cases if they included a positive diagnostic test result and chart review revealed clinical illness consistent with the test result. Written informed consent was obtained from parents or legal guardians. Institutional review board (IRB) approval was obtained prior to the initiation of the study.

Initial sampling coincided with admission blood collection and was drawn before IVIG treatment (if applicable). Generally, 5–10 milliliters of blood was drawn up in sodium heparin tubes and placed at room temperature on a neutator in the locked clinical laboratory in the emergency department. Attempts were also made to collect samples 48–72 hours (post- IVIG treatment when applicable) after first blood draw for those who were admitted.

Notable exclusions to prevent effects of excessive blood draws included prior study enrollment within two months, chronic or active blood borne infection (*i*.*e*. human immunodeficiency virus (HIV), hepatitis B or hepatitis C virus (HCV)), chronic anemia, excessive blood loss or other excessive blood taken for lab studies in the last eight weeks. Demographic and laboratory information collected included duration of fever, age, race, additional symptoms, laboratory values, and microbiology results. Pertinent exam findings such as patients’ vital signs, and physician’s evaluation were also documented. Access to the medical record was included in the consent to allow for review of microbiology and other laboratory data.

### Isolation of peripheral blood mononuclear cells (PBMCs)

Generally, we followed established published protocols [1]. Blood was collected in sodium heparin tubes and placed on a neutator until collection within 1–15 hours. Samples were processed in a BSL-2 biosafety cabinet. Blood was initially mixed 1:1 with phosphate buffered saline (PBS) (Gibco by Life technologies. Carlsbad, CA). This was layered onto a polysaccharide solution (Ficoll-paque. Sigma, St. Louis, Missouri) and centrifuged at 400 x G for 25 minutes in a containment bench top centrifuge with locking top. 1:1 PBS to Plasma samples were collected from the top layer and stored at -80 C° for future studies. PBMCs were then withdrawn from the meniscus layer, transferred to another tube and washed twice with 10% Fetal bovine serum (FBS) (Media Tech, Corning. Manassas, Virginia). Cells were then counted and cryopreserved with 5–10 million cells per one mL in 10% DMSO and 90% FBS freezing medium.

### Isolation of B cells

**Fig 1:**
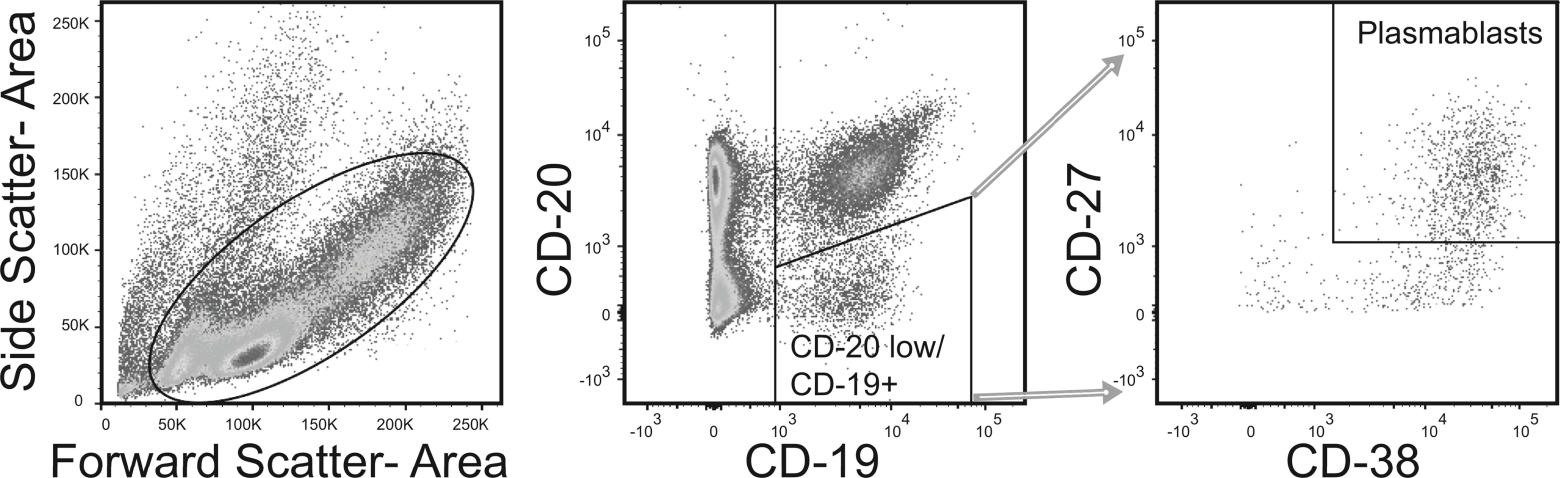
Flow cytometry isolation of plasmablasts. A single example of the flow gating and analysis is shown. After selecting for single cell events, lymphocyte gate was constructed inclusive of blasting cells, which are enriched for plasmablasts. CD14+ and CD3+ cells were excluded. CD19+/CD20low cells that were dual positive for CD27 and CD38 were defined as plasmablasts.

Cells were removed from cryopreservation and thawed in 37°C water bath. Cells were repetitively washed with 10% FBS in PBS. On the final dilution, cells were diluted to 1x10^6^ cells/mL of 2% FBS in PBS. Cells were labeled with Fluorophore-conjugated goat monoclonal antibodies to the following human antigens; CD3, CD14, CD19, CD20, CD27, CD38 and IgG; purchased from Becton Dickinson Bio-sciences (San Jose, CA, U.S.A.) and with IgA+ purchased from Miltenyi (Bergisch Gladbach, Germany). Cells were labeled in 2% FBS/PBS and washed twice with 2% FBS/PBS prior to flow cytometric analysis. Flow cytometric analysis was performed with a FACSAria flow cytometer in a Biosafety Level 2+ laboratory aerosol containment accessory (Becton Dickinson, Franklin Lakes, NJ). Plasmablasts were separated similar to previous published studies [24, 44] (Fig 1).

### ELISA measurement of Interleukin-21

Methods follow those previously described [45]. Manufacturer’s protocol for the Human IL-21 ELISA kit (Ready-SET-Go! Kit, Affymetrix, San Diego) with a published sensitivity range of 8–1000 pg/mL was used in this study. Anti-Human IL-21 capture antibody was bound to 96-well ELISA plates overnight (o/n) at 4°C. IL-21 coated plates were washed three times (3x) then blocked (1 hr, room temperature (RT)), then washed once. Human IL-21 internal standards (ranging from 15.625 pg/mL to 800 pg/mL) and diluted plasma samples were layered on the plate in duplicate and incubated (o/n, 4°C). After washing (5x) plates were incubated (1 hr, RT) with anti-Human IL-21 Biotin detection antibody. Plates were washed (5x) and incubated (30 min, RT) with Avidin-HRP enzyme. After washing (7x), plates were developed with TMB solution with 2N H_2_SO_4_ acid stop and read at 450 nm in a spectrophotometer. Plasma was initially diluted with PBS during PBMC isolation (to roughly 40% of full concentrated plasma) and this was factored in to final IL-21 level reported.

### Data analysis

Descriptive characteristics for study subjects were computed after detailed chart review. Categorical variables were reported as proportions in percentage, and continuous level variables were reported as means and standard deviations. Chi-square analysis was used to compare differences in level of white blood cells in urine among KD and control patients. Separate independent t-tests were used to examine differences between patients with Kawasaki Disease and controls for variables that included fever, white blood cell count, platelet count, C-reactive protein (CRP), Sedimentation rate (ESR), hemoglobin, hematocrit, Aspartate aminotransferase (AST), Alanine aminotransferase (ALT), and serum albumin. Flow cytometry data was analyzed using FlowJo software (Ashland, Oregon). Statistical tests, as described in the text, were two-tailed with alpha of 0.05 and performed using Prism software (Graphpad, La Jolla, CA).

## Results

### Clinical comparison

Patient ages, gender and discharge diagnosis are listed in Table 1. A number of associations that are commonly described with KD [46] are notable: one individual on presentation fulfilled echocardiography criteria, four individuals had hydropic gallbladders, one child had a retropharyngeal phlegmon, one child had recurrent KD, and four children had imaging of their adenopathy consistent with clustering of nodes, rather than a single inflamed node. All laboratory values and samples from KD children analyzed were drawn prior to IVIG therapy unless noted in the text. Analysis of laboratory values between KD and total febrile controls showed relative thrombocytosis, hypoalbuminemia, anemia, and elevation of inflammatory markers in KD children (Table 2), which is similar to previously published studies related to KD [47–49]. Notably, control subjects had a relative elevation of Aspartate Aminotransferase (AST) in our cohort.

**Table 1:** Clinical characteristics of enrolled patients, classified by diagnoses.

Enrolllee #	Gender	Age months	Febrile days before blood drawn	Clinical Diagnosis and/or associated symptoms and diagnostic result
**Kawasaki Disease (KD)**				
4	female	69	7	Kawasaki Disease
5	male	32	7	Kawasaki Disease, Recurrence of diagnosis 3 months prior
6	male	28	6	Kawasaki Disease, Gallbladder hydrops
15	male	78	6	Kawasaki Disease
17	male	49	4	Kawasaki Disease, Polyarthritis
24	male	58	4	Kawasaki Disease, neck ultrasound with node cluster
47	female	81	5	Kawasaki Disease, + Parainfluenza 2 Respiratory Screen
53	male	29	6	Kawasaki Disease
67	male	10	6	Kawasaki Disease
68	male	21	4	Kawasaki Disease
75	male	20	7	Kawasaki Disease, +Rapid Strep screen, + Echo (lack of tapering, LAD z score 2.5), Gallbladder hydrops, neck ultrasound with node cluster
79	male	26	7	Kawasaki Disease
80	female	53	3	Kawasaki Disease, neck CT with node cluster, retropharyngeal phlegmon
93	female	83	5	Kawasaki Disease, Gallbladder hydrops, neck ultrasound with node cluster
97	male	54	6	Kawasaki Disease, + RSV Respiratory Screen, Gallbladder hydrops
99	female	39	9	Kawasaki Disease
103	male	23	5	Kawasaki Disease
119	male	29	6	Kawasaki Disease
**Febrile Controls**				
**Febrile Controls: Prolonged Fevers**				
54	male	71	14	Septic Arthritis, pretreated
66	male	40	14	Rash, Conjunctivitis
94	female	42	24	URI, Conjunctivitis (+ Adenovirus)
123	male	30	14	URI, Adenopathy
**Febrile Controls: Adenoviral infection**				
45	female	72	5	URI, Rash, conjunctivitis
59	male	36	6	URI, Rash, conjunctivitis
95	female	75	5	URI, Rash, conjunctivitis
131	male	19	4	URI, conjunctivitis
**Febrile Controls: Influenza**				
49	male	36	2	Rapid Influenza A+, Rash
101	female	85	3	Rapid influenza A + (OSH)
109	male	32	2	Rapid influenza B + (OSH)
**Febrile Controls: Hand-foot-and-mouth disease (HFM)**				
16	female	21	3	Hand-foot-and-mouth disease (HFM)
87	male	35	5	Hand-foot-and-mouth disease (HFM)
104	male	28	6	Hand-foot-and-mouth disease (HFM)
117	female	34	4	Hand-foot-and-mouth disease (HFM)
**Febrile Controls: Skin and Soft Tissue Infections (SSTI)**				
7	male	13	4	MRSA Abscess, Cervical adenitis
25	male	37	2	Cellulitis of the leg
30	female	13	3	Periorbital cellulitis
42	male	15	2	Cervical adenitis; improved on clindamycin
43	male	50	4	Parapharyngeal abscess (*S*. *viridans*)
48	male	14	11	Cervical adenitis; improved on clindamycin
60	male	37	7	Cervical adenitis, conjunctivitis; improved on clindamycin
98	male	14	5	Cervical adenitis, Rash, Conjunctivitis; improved on ampicillin-sulbactam
107	male	88	10	Cervical adenitis; improved on clindamycin
130	male	52	7	Cervical adenitis; improved on clindamycin
**Febrile Controls: Group A Streptococcal pharyngitis (GAS)**				
58	male	68	9	Pharyngitis, Cervical adenopathy, Rapid strep +
89	male	62	4	Pharyngitis, Rash, Rapid strep +
92	female	20	2	Pharyngitis, Rapid Strep +
113	male	34	3	Pharyngitis, Scarlet fever, Rapid Strep +
**Febrile Controls: not otherwise classified**				
3	male	19	4	URI, Rash
10	male	56	4	Viral syndrome, bilateral adenopathy
12	male	27	6	Viral pneumonia
13	female	64	3	Viral pneumonia, Gastroenteritis, Conjunctivitis; + Parainfluenza 3 Respiratory Screen
14	male	22	1	Cough
20	female	14	6	URI, Rash
29	female	52	6	Pyelonephritis, Septicemia
31	female	15	5	Viral syndrome, Neutropenia
34	male	23	7	Bacteremia (*Salmonella*), Enteritis
52	male	80	3	Bacteremia (*S*. *pyogenes*)
55	male	11	7	Acute pyelonephritis (*E*. *coli*)
61	male	21	5	Cough, Rash, Conjunctivitis
64	female	82	6	Viral Syndrome, Peeling
65	male	18	2	Viral Syndrome
69	female	27	6	Rash
70	male	9	4	URI
71	female	16	3	Viral syndrome, Rash
72	male	108	5	Viral syndrome, Hepatitis, Rash, Conjunctivitis
74	male	12	7	Viral syndrome, + Enterovirus
76	female	38	2	Allergic reaction with fever, Rash, Hand and feet swelling
77	male	48	4	Viral syndrome, Rash, Allergic drug reaction
78	male	67	2	Viral syndrome, Adenopathy
81	male	11	5	Viral syndrome, Rash
82	male	46	2	URI, Conjunctivitis, Adenopathy
83	male	14	6	Gastroenteritis, Rash
84	male	23	4	URI, Gastroenteritis, Rash
85	female	16	1	Viral Syndrome, Rash
86	female	49	4	Viral syndrome, Gastroenteritis
88	male	43	2	URI
90	female	43	6	Viral syndrome, Gastroenteritis
91	female	57	6	Viral Pharyngitis
102	male	10	1	URI, Cough
106	male	29	2	Erythema multiforme
110	male	59	1	Staph scalded skin syndrome
112	male	11	7	Gastroenteritis
115	female	40	4	Rash, Conjunctivitis
116	male	11	6	Viral syndrome, Rash
122	male	16	3	Rash
125	male	12	3	Rash, Conjunctivitis, referred to rule-out KD
129	female	10	1	URI, Conjunctivitis

**Table 2:** Demographic and laboratory comparison of Kawasaki disease to control.

Variable	Kawasaki Disease (KD) (n = 18)	All Controls (n = 69)	P-value
Male Gender, n (%)	13 (72.2)	49 (71.0)	0.920
Age in months ±(SD)	43.4(23.0)	36.3 (23.6)	0.955
Febrile days prior to blood draw ±(SD)	5.7 (1.4)	5.0 (3.7)	0.208
White blood cell count maximum ±(SD)	17.6 (5.4)	13.1 (6.0)	***0*.*005***
Platelet count maximum ±(SD)	393.4 (113.2)	326.7 (145.9)	***0*.*046***
C-reactive protein [CRP] maximum ±(SD)	129.4 (62.3)	73.3 (88.9)	***0*.*040***
Sedimentation rate [ESR], prior to IVIG ±(SD)	83.6 (30.3)	55.4 (28.4)	***0*.*005***
Hemoglobin minimum ±(SD)	10.4 (1.2)	11.6 (1.0)	***<0*.*001***
Hematocrit %, minimum ±(SD)	31.2 (3.5)	34.8 (2.9)	***<0*.*001***
Aspartate Aminotransferase [AST] maximum ±(SD)	36.8 (20.0)	52.4 (31.2)	***0*.*042***
Alanine Aminotransferase [ALT], maximum ±(SD)	51.2 (36.5)	57.1 (78.3)	0.726
Serum Albumin minimum ±(SD)	2.9 (0.5)	3.7 (0.5)	***<0*.*001***
Urine WBC, n (%) 0–5 6–100	6 (40) 9 (60)	22 (78.6) 6 (21.4)	***0*.*011***

### Peripheral B cells

**Fig 2:**
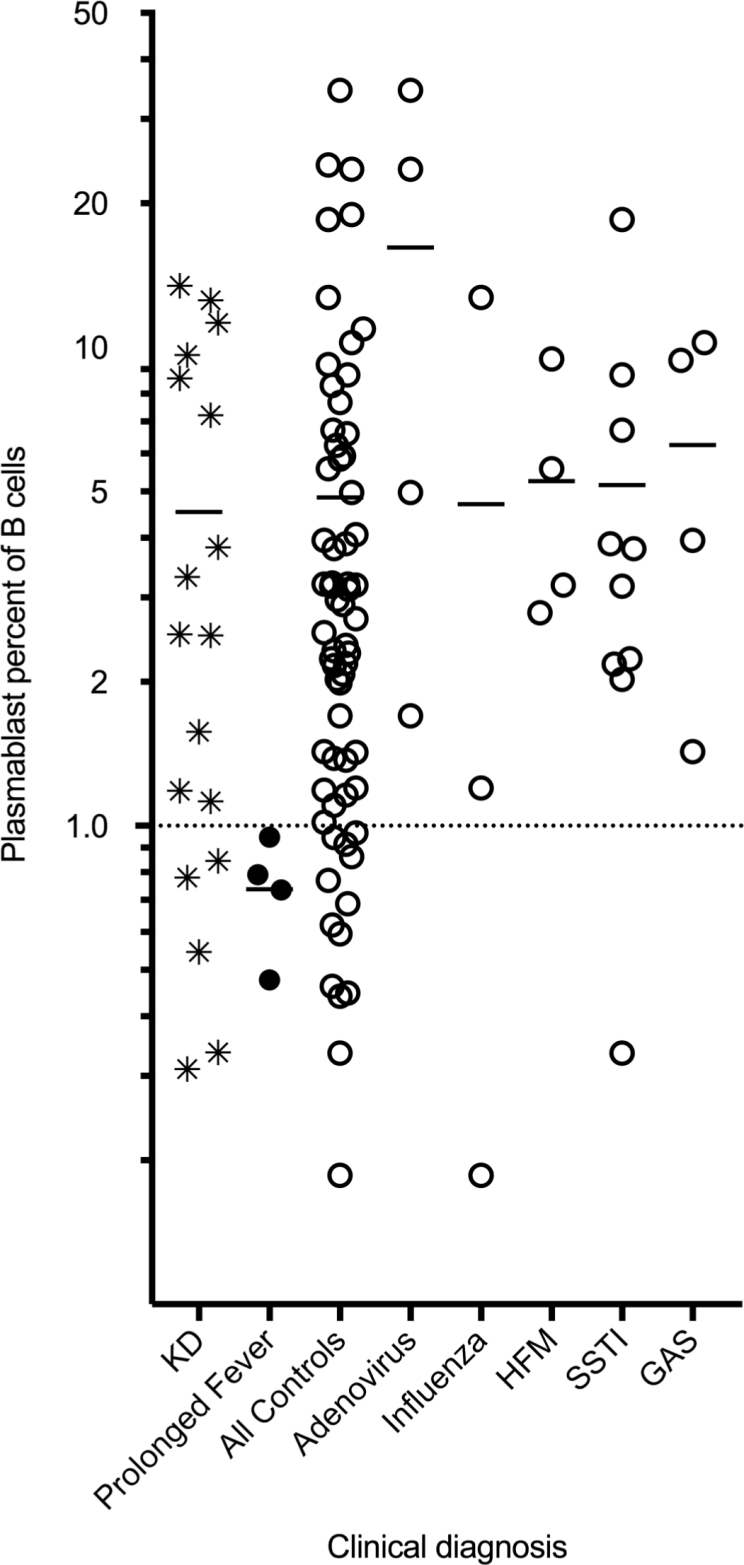
Plasmablast comparison between different clinical diagnoses. Plasmablast levels, as a percentage of overall B cell number, were compared between children with KD (star), prolonged fever (closed circle), and all controls (open circles). Mean values are marked by horizontal dash. Data is shown on a logarithmic scale to improve separation of individual points. Five subsets of all controls that qualified as specific diagnoses are shown (Adenovirus, Influenza, Hand-foot-and-mouth (HFM), Skin and Soft tissue infections (SSTI), and Group A streptococcal pharyngitis (GAS)) and also listed in Table 1.

Plasmablast levels showed no significant differences between KD and our cohort of febrile controls (medians/means in our KD and control groups were 2.51%/4.53% and 2.32%/4.86% of B cells respectively with ranges in KD patients of 0.31% to 13.45%, and ranges of controls 0.18% to 34.44%) (Table 3 and Fig 2). In people without recent infection or in convalescence, circulating plasmablast levels are generally less than 1.0% and in some studies nearer to 0.40% of circulating B cells [24, 25]. In our cohort, four children had prolonged fever (>14 days) and their levels are consistent with background levels previously published (mean = 0.74 and median = 0.75, Fig 2 closed circles). Subsets of control cases with less than two weeks of fevers that qualified for specific diagnoses are also shown (open circles, Fig 2). These include five subgroups: confirmed cases of adenovirus, influenza and group A streptococcus pharyngitis (GAS) as well as groups of primarily clinical diagnoses of hand-foot-and-mouth disease and bacterial skin and soft tissue infections (SSTI). When evaluating for elevation over background, both KD and febrile control plasmablast percentage were significantly elevated when compared to our prolonged fever cohort, (p values of 0.0329 and 0.0079 respectively). The only difference of KD to any other group was the notable elevation within the adenoviral group.

**Table 3:** Median values of cell subsets from flow cytometry of peripheral blood mononuclear cells.

Cell Subset		Kawasaki (n = 18) Median value 2.5–97.5^th^ Percentile	Control (n = 69) Median value 2.5–97.5^th^ Percentile	P value
Percent of Lymphocytes	B cells	17.3 15.1–22.9	14.8 14.1–17.5	0.081
	IgG Cells	1.19 0.94–1.53	0.71 0.71–1.01	***0*.*005***
	IgA Cells	0.69 0.57–1.48	0.56 0.57–0.92	0.237
	Plasmablast	0.33 0.33–1.10	0.29 0.45–0.98	0.607
Percent of B cells	IgG	7.15 5.56–9.77	4.74 4.85–6.44	0.074
	IgA	4.35 3.69–7.90	3.59 3.81–6.10	0.470
	Plasmablasts	2.51 2.24–6.82	2.32 3.11–6.14	0.942
Percent of Plasmablasts	IgG	11.93 9.96–15.34	9.24 9.00–12.16	0.177
	IgA	47.4 36.6–54.2	43.6 38.14–45.51	0.441

In KD children, we also noted a similar elevation of B cells consistent with previous publications [6, 14, 19, 50], and in particular, an elevation of the IgG+ B cell subset of total lymphocytes reaches significance (p = 0.005). We also looked specifically at IgA+ B cells as these have been implicated in specific systemic immune responses with subsequent infiltration of the aneurysmal tissues [51, 52] and shown to be low in a single previous studies [1]. There was no difference in the overall numbers of IgA+ B cells between our KD and control subjects.

**Fig 3:**
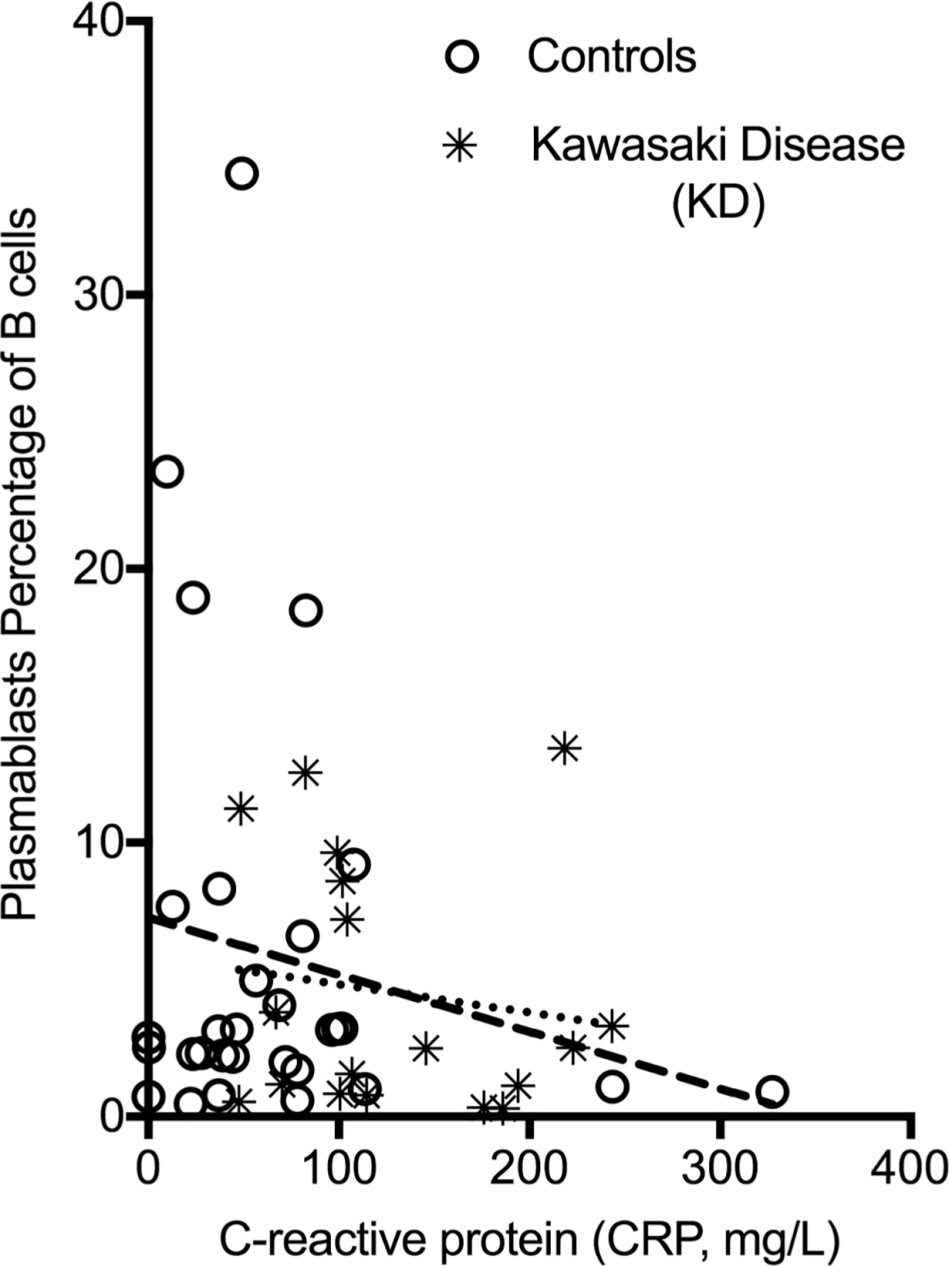
Plasmablast level relationship to C-reactive Protein (CRP). Linear regression analysis of level of CRP and plasmablast level in KD (starred, small dashed line) and controls (open circles, long dashed line). Results failed to show any linear correlation.

Since plasmablasts can also be highly elevated during autoimmune phenomenon, and levels are shown to correlate with disease flares in these cases [36–40], we chose to explore if there was a correlation of overall inflammation to plasmablast level in our cohort. All 18 of our KD samples and 48 of our controls had CRP data available for analysis. Unlike what has been described for a number of autoimmune conditions, there is no significant correlation of CRP and plasmablast responses in either controls or our KD samples (R^2^ values were <0.1). Slopes of the linear regression lines actually implied negative correlation of CRP to plasmablast level, but they were not significantly different than zero (Fig 3).

**Fig 4:**
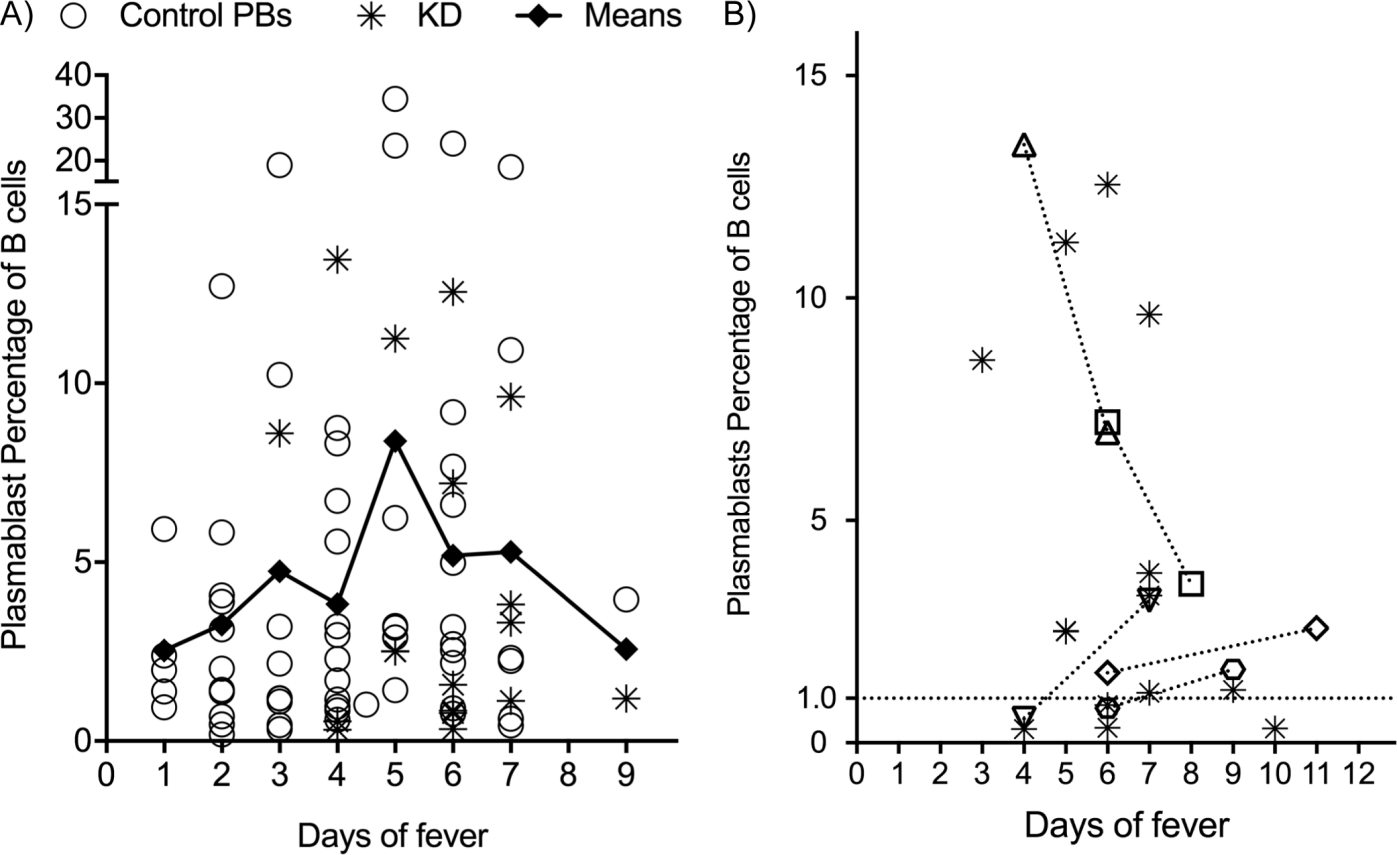
Relationship of plasmablast level to day of fever. A) Values of means of all samples graphed by day (diamonds) with connected line showing trend. KD samples (stars) and controls (open circles) are shown. B) Showing KD samples alone, single timepoint samples are again labelled with stars. Samples of five individuals with repeat samples are shown by connected (short dash) lines and distinguished by open distinct symbols.

Notably, a number of KD and control children had non-stimulated plasmablast levels, which may be due to a number of factors. First, we evaluated if presentation day of fever of the samples analyzed were different in these subjects. We only had a select few individuals with multiple blood samples, so we chose to pool data by day of fever, similar to previous publications on meningococcal vaccination [26] and RSV infection [42]. Each individual underwent a review of the medical record to assign the day of fever associated with each sample. Overall, the peak in both KD and control cohorts (days 3–7) is consistent with reports of other infectious diseases [24, 26, 35] (Fig 4A). KD samples mainly had elevations on day 4–6 with peak mean was on day 5. Even in this peak time, there was a wide variability in plasmablast level. Five KD patients had paired samples taken 48–72 hours later after the initial blood draw and after initiation of IVIG therapy. No consistent trends were seen. Analyzing KD samples with multi-timepoint blood sampling (Fig 4B), shows that three initially low values rose, while two plasmablast levels that were high on day 4 and 5 receded over time.

**Fig 5:**
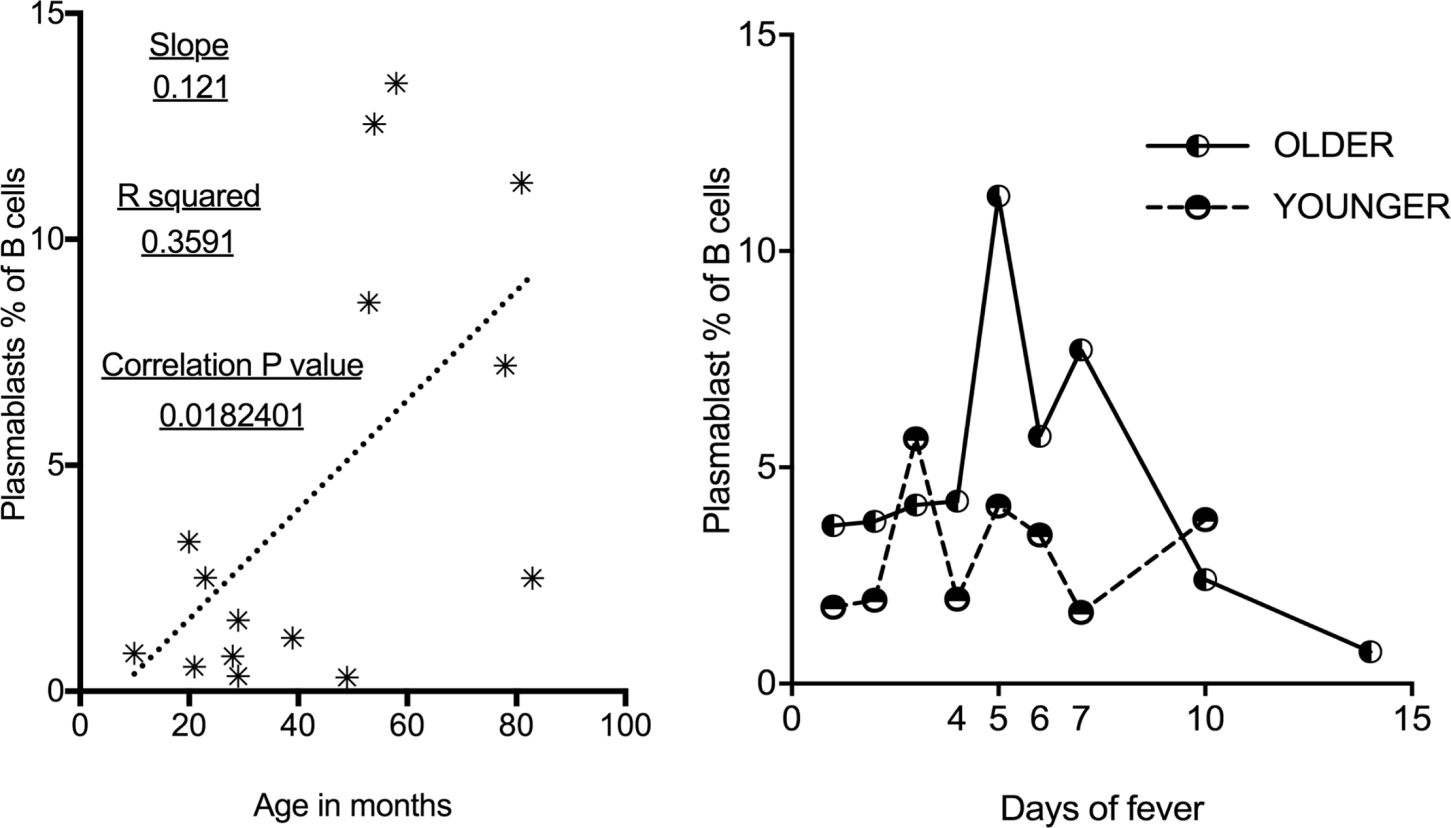
Plasmablast level and temporal pattern relative to age of subject. A) In KD children, Linear regression (small dashed line) shows relationship of older children to higher plasmablast levels. B) Mean values for plasmablast percentage of B cells by day of fever for those 24 months and under (long dashed line) compared to subjects over 24 months of age (solid line).

The age of each individual may affect plasmablast levels in direct or indirect fashion. Younger children are more likely to show naïve immune reponses to infection and this has been shown to potentially temporally delay the rise of plasmablasts [34]. Immune defects are described in young children that may prevent robust responses to vaccines and infections [53]. Since our cohort included both infants and toddlers, we evaluated plasmablast responses in relation to the age of the subject. Exploring the effect of age on plasmablast levels shows that overall, younger children have diminished plasmablast responses. Linear regression analysis of age in months compared to plasmablast levels of KD, controls, and all samples show slopes that are all significantly non-zero (0.018, 0.015 and 0.003 respectively) with generally higher values in older children. R^2^ values for these groups are 0.359, 0.149 and 0.161 respectively. Data for the KD group is illustrated in Fig 5.

Comparing plasmablast levels in younger children (0–24 months) to older children (25 months and greater; unpaired t-test, Welch’s correction for variable Standard Deviations), indicated that younger children had significantly (p<0.024) lower circulating plasmablast numbers (means and SEM of 2.99 ± 0.63 versus 5.59 ± 0.93; Standard Deviations of 3.62 and 6.87 respectively). Comparing pooled data from days 4–7 (the described peak for a number of viruses in the literature) also shows a significant difference (p <0.007) between these age groups (up to 24 months old n = 20 and greater than 24 months of age n = 33; Mean and SEM of 2.82 ± 0.5931 and 7.097 ± 1.395, and Standard Deviations of 2.65 and 8.02 respectively). This is illustrated by comparing mean results by day in these two age groups (Fig 5B). This supports the theory that there is an inherent blunting of this response overall in young children, although other physiologic phenomenon beyond purely age may be playing a role (see discussion).

There are a number of potential immune differences that may explain this. One factor that supports plasmablast formation [54] and has been previously associated with KD [55] is IL-21. We previously attempted to confirm this association when comparing KD to febrile controls [45], however we did not explore age effect or relationship to plasmablast level previously. Here, we further analyzed IL-21 levels previously obtained in the context of age in a subset of the children in this study (13 Kawasaki children and 36 controls). On regression analysis of IL-21 related to either age in months or plasmablast % of B cells, slopes were not significantly different from zero. R^2^ for KD versus controls for these groups were <0.1 (0.055 and < .001 for plasmablast and IL-21 and 0.022 and 0.025 for plasmablast and age in months).

## Discussion

Overall, this work reveals initial observations on overall poor plasmablast response in children at young ages. Generally, it has long been known that infants have a less ‘mature’ immune system than adults. Young children tend to have anti-inflammatory (TH2) skewing of their responses and poor humoral immunity [56]. Diminished express of the plasma cell survival factor, APRIL, has been shown previously to contribute to poor humoral immunity in both bone marrow [57]. Our results imply even before reaching the bone marrow, there is a significant issue in generation of the plasma cell precursors. This can be explained by poorer germinal center function, lower T-cell follicular helper cells and weaker B-cell receptor signaling shown predominantly from murine studies [58]. The choice of vaccine adjuvant may help overcome this anti-inflammatory skewing in children [59] and could improve these plasmblast numbers, but this specific question has not been addressed in the published studies on these alternative adjuvants. Although our results are consistent with what is physiologically known about poor germinal center responses, plasmablast responses have not been extensively explored in human studies. Only limited studies on meningococcal vaccines have collected samples at different timepoints to explore plasmablast temporal development. Overall, infants had a lower peak response [42] compared to historical data collected from adolescents utilizing the same vaccine [43].

B cells are obviously important for adequate response to infectious diseases, but a number of studies support a role for B cell responses in KD as well. Genome wide association studies have identified single polymorphisms in B-cell lymphoid kinas (BLK) and CD40 that correspond to disease risk for KD [21]. The results of our study are consistent with the majority of the literature that show B cell stimulation and increasing peripheral B cell numbers during KD [6, 14, 19, 50]. Previous post-mortem pathology studies of aneurysms have shown IgA+ plasma cell infiltrates within the coronary arteries of patients with KD [51] and have shown these are predominantly oligoclonal responses [52]. However, studies on circulating IgA+ cell numbers have shown mixed results. [1, 5]. Notably, we did not see an increase in circulating IgA+ B cells. In these studies on plasma cell infiltrates, the autopsy specimens were generally from convalescent timepoints so may not reflect acute illness changes. Potentially, the IgA+ cell infiltrates solely represent a non-specific inflammatory state, such as seen with other IgA infiltrating plasmas cells in NMDAR encephalitis [60], primary sclerosing cholangitis [61], multiple sclerosis [62], and rheumatoid pericarditis [63].

As plasmablasts are enriched for antibodies against the challenging antigen in a number of infections and in vaccinations, we are interested to explore if they can be used to identify the unknown causes of such disorders as Kawasaki disease. For this, our study raises a number of issues. We did observe a variable plasmablast response in KD children which could be seen for a number of reasons. Even on paired sampling, we show a rise in plasmablasts in three of five samples on the second blood draw. Assuming a single etiology sets off KD, potentially, the plasmablast response of the associated infection of KD may have a more unpredictable range than many other infections described [35]. Respiratory Syncytial Virus in particular shows a wide variance over time compared to other infections [26]. Another possibility would be that exposure to the infection may show differences in a naïve and a memory response, such as seen with Rabies vaccination in adults [34]. The fact that there is elevation of peripheral IgG+ cells in our cohort also implies a memory response. However, this would contradict the theory of protection by maternal immunity and that the age of KD incidence implies protection after the first “infection”. Additionally, antibodies cloned from plasmablasts during chronic HIV infection shows polyreactivity and poor enrichment [31, 32]. Polyspecificity is not unique to HIV as it has been shown in studies on acute dengue virus [25] and salmonella infections [29]. If KD is set off by a particular virus that has a lower level of plasmablast enrichment for specific antibodies, then identifying one or several KD specific antibodies may prove very challenging.

Considering that it may not be just a single infection, multiple infectious agents with different afebrile prodromes may lead to the KD state. We did not specifically enroll patients in a time course nor recruit on specific days after the beginning of fever, so our data may not overlap with the optimal window to see the plasmablast rise in all patients if this is set off by a variety of infectious agents. Also, viral infections without as much systemic viremia, such as RSV, show overall less responsive and predictable plasmablast peaks [26]. If this type of infection sets off KD, that would account for the large ranges in both groups of patients and explain many of the normal values of plasmablasts seen in both groups.

Two other notable observations are worth mentioning. First, the effect of IVIG on plamasblast responses are unknown. We only have limited data (five paired sample before and after) and with the mixed effects seen (see Fig 4B), it is unclear if a conclusion can be drawn. For future timecourses of KD children, this may be a significant confounder. Secondly, in our control group, the subjects positive for adenovirus has some of the highest levels of plasmablast percentage as a group in our cohort when compared to other described infectious diseases [28]. Studies on plasmblast responses in adenovirus in children have not been previous published, but the response appears similar to the robust responses seen during dengue infection [28]. This did not exclusively account for the highest levels as three other individuals also had peripheral circulating plasmablast percentages >15%. Although dengue virus was not tested for in these samples, there was no travel history or clinical consistency in our cohort. Future studies on a larger group of adenovirus infected children would be of interest. As mentioned, most studies have been done in adults, so children of certain ages (outside of infancy) may actually have a more robust potential to have this type of significant elevation. Although intriguing, there are too few samples to draw firm conclusions at this time.

## Conclusions

This work reveals initial observations on plasmablast responses from natural infections in children at young ages. The similar plasmablast responses shown further support a role for an infectious disease setting off the inflammatory cascade of KD. Future studies evaluating plasmablasts age effects and naïve versus memory responses by repeated sampling over time are warranted to further clarify the normal responses in young children. This will assist in future studies on optimizing vaccine adjuvants in these ages. The similarity of plasmablast responses shown here implies that circulating plasmablasts during KD produce antibodies that specifically target the etiology of Kawasaki disease. Although this might prove difficult for the reasons outlined, further description of the dynamism of these cell levels and studies on the antibodies they express offers the hope of improved diagnostic tests or, potentially, a path to identify the etiology of KD.

## Supporting information

S1 Dataset Complete demographic, clinic, and experimental value dataset. This dataset contains all raw values that contributed to the analyses within this manuscript organized by diagnosis. Raw flow cytometry data and data on Interleukin-21 levels are included as well as data and calculations highlighted in Tables 1–3 and Figs 1–5.
(XLSX)
